# Experiences and outcomes of maternal Ramadan fasting during pregnancy: results from a sub-cohort of the Born in Bradford birth cohort study

**DOI:** 10.1186/1471-2393-14-335

**Published:** 2014-09-26

**Authors:** Emily S Petherick, Derek Tuffnell, John Wright

**Affiliations:** Bradford Institute for Health Research, Bradford Teaching Hospitals NHS Trust, Bradford, BD9 6RJ UK; School of Health Studies, University of Bradford, Bradford, BD7 1DP UK; Bradford Teaching Hospitals NHS Foundation Trust, Bradford, BD9 6RJ UK

**Keywords:** Ramadan, Fasting, Pregnancy, Preterm delivery, Birth weight

## Abstract

**Background:**

Observing the fast during the holy month of Ramadan is one of the five pillars of Islam. Although pregnant women and those with pre-existing illness are exempted from fasting many still choose to fast during this time. The fasting behaviours of pregnant Muslim women resident in Western countries remain largely unexplored and relationships between fasting behaviour and offspring health outcomes remain contentious. This study was undertaken to assess the prevalence, characteristics of fasting behaviours and offspring health outcomes in Asian and Asian British Muslim women within a UK birth cohort.

**Methods:**

Prospective cohort study conducted at the Bradford Royal Infirmary UK from October to December 2010 comprising 310 pregnant Muslim women of Asian or Asian British ethnicity that had a live singleton birth at the Bradford Royal Infirmary. The main outcome of the study was the decision to fast or not during Ramadan. Secondary outcomes were preterm births and mean birthweight. Logistic regression analyses were used to investigate the relationship between covariables of interest and women’s decision to fast or not fast. Logistic regression was also used to investigate the relationship between covariables and preterm birth as well as low birth weight.

**Results:**

Mutually adjusted analysis showed that the odds of any fasting were higher for women with an obese BMI at booking compared to women with a normal BMI, (OR 2.78 (95% C.I. 1.29-5.97)), for multiparous compared to nulliparous women(OR 3.69 (95% C.I. 1.38-9.86)), and for Bangladeshi origin women compared to Pakistani origin women (OR 3.77 (95% C.I. 1.04-13.65)). Odds of fasting were lower in women with higher levels of education (OR 0.40 (95% C.I. 0.18-0.91)) and with increasing maternal age (OR 0.87 (95% C.I. 0.80-0.94). No associations were observed between fasting and health outcomes in the offspring.

**Conclusions:**

Pregnant Muslim women residing in the UK who fasted during Ramadan differed by social, demographic and lifestyle characteristics compared to their non-fasting peers. Fasting was not found to be associated with adverse birth outcomes in this sample although these results require confirmation using reported fasting data in a larger sample before the safety of fasting during pregnancy can be established.

**Electronic supplementary material:**

The online version of this article (doi:10.1186/1471-2393-14-335) contains supplementary material, which is available to authorized users.

## Background

Annual fasting during the holy month of Ramadan is one of the five pillars of Islam. During Ramadan all able bodied Muslims abstain from food, fluids, smoking and oral medications between sunrise and sunset [1]. Muslim women, pregnant during Ramadan, are exempt from fasting if they are concerned about their health, or the health of their baby as are persons with pre-existing health conditions. Despite this dispensation many women continue to observe the fast throughout pregnancy. Concerns have been raised about the potential health impacts on the developing child although the evidence of detrimental effects on both birth weight and gestational length in babies exposed to fasting in utero has been inconsistent [2–4].

Contemporary evidence on Ramadan fasting behaviours undertaken by pregnant women in the UK is scarce, with the last survey published in 1982 [5]. As objective measurement of fasting is difficult, it remains unclear if fasting behaviours are consistent across populations, particularly between Western and majority Muslim countries. There is potential for bias to have been introduced to results of studies examining fasting and health outcomes if the exposure of fasting is not homogenous amongst pregnant Muslim women but rather differs by other factors associated with health outcomes.

Recent data from the 2011 UK census shows that the Muslim population of the UK now comprises 4.8 per cent of the population up from three per cent in the previous 2001 census and locally in Bradford 24.7 per cent of the population reports their religious group as Islam [6]. Given these demographic changes in the UK we require an updated knowledge of fasting behaviours of UK pregnant women and contemporary evidence of health outcomes.

We used data from a sub cohort of the Born in Bradford (BiB) birth cohort study to explore the Ramadan fasting behaviours of pregnant Muslim women and associated birth outcomes.

## Methods

### Study setting

Born in Bradford (BiB) is a longitudinal multi-ethnic birth cohort study investigating the impact of environmental, psychological and genetic factors on maternal and child health and wellbeing [7]. Bradford is a city in the North of England with high levels of socio-economic deprivation and ethnic diversity. Approximately half of the births in the city are to mothers of South Asian origin. For those consenting, a baseline questionnaire was completed via an interview with a study administrator. The BiB cohort recruited 12,453 women during 13,776 pregnancies between 2007 and 2010 and the cohort is broadly characteristic of the city’s maternal population [7]. The participants gave informed consent for the data collection and ethical approval for the data collection was granted by Bradford Research Ethics Committee (Ref 07/H1302/112).

### Study population

Between October and December 2010, a consecutive sample of 310 pregnant women who reported their ethnicity as Asian or Asian British and reported their religion as Islam were approached and agreed to participate in this study. We included results from all women who had live singleton births with a linked maternity record resulting in an eligible sample of 300 women for this study. Ten women were excluded from the analysis, n = 1 did not have linked birth information for, n = 7 excluded as had multiple births, n = 2 did not have a live birth.

### Fasting behaviour

In addition to the baseline questionnaire, participants in this sub study were asked two extra questions relating to their fasting behaviour during the preceding Ramadan. In 2010, Ramadan occurred between 11^th^ of August to 8^th^ of September, a total period of 29 days with fasting times of up to 18 hours per day. The first question asked women if they had fasted during Ramadan in their pregnancy this year; response options were (fully- full 29 day period), yes (partly- any but not the total 29 day period) or not fasting and secondly asked pregnant women how many days they had fasted. We explored the effect of fasting by grouping fasting behaviour into a binary, fasted not fasted classification. To test for a dose response effect of fasting we also examined the effect of fasting as a categorical variable grouped as none, 1–10 days, 11–19 days, 20–29 days.

### Outcomes

The primary outcome was fasting behaviour, defined as fasting or not fasting. Secondary outcomes of this study were preterm delivery and birth weight as they had previously been reported to be associated with Ramadan fasting [2–4, 8]. Preterm delivery (PTD), was defined as birth before gestational week 37 + 0. Gestational length defined as length of gestation after amenorrhea (in weeks), which was based on last menstrual period date confirmed by dating ultrasound conducted at 12 weeks gestation. If there were less than seven days difference between these two dates the last menstrual period date is used for the estimated date of delivery, whereas if there is a greater than seven days difference then the ultrasound dating scan is used to assign the estimated date of delivery.

### Covariables

A priori we selected covariables that are known to be associated with the outcomes of interest and/or hypothesised to be associated with the decision to fast. These included trimester exposed to fasting, booking BMI (categorised as underweight <18.5, normal > =18.5-24.9, overweight > =25-29.9, obese > =30), maternal age (mean centred), parity (0,1,2,3+), ethnic origin (Pakistani, Indian, Bangladeshi or other South Asian), marker of acculturation (born in the UK or migrated to the UK aged five or less vs migrated to the UK aged greater than five years), smoking status (yes or no), employment status (currently worked, ever worked or never worked), marital status (married, single or divorced/widowed), maternal education (<5 General Certificate of Secondary Education (GCSEs) qualification, 5 GCSEs (*standard minimum level of education when leaving school*), A level equivalent, Higher than A level, Other), mother living in an extended family (yes or no) and consanguinity (in consanguineous relationship vs not in consanguineous relationship). We also explored markers of health during pregnancy including gestational and pre-existing diabetes, pregnancy induced hypertension (defined as having a blood pressure higher than 140/90 measured at two or more periods at least 6 hours apart) and pre-eclampsia (defined as defined as proteinuria (+0.3 gms with blood pressure >/=140/90 after 20 weeks of pregnancy on more than one occasion) which were all categorised as yes or no to ascertain whether women with health problems took the option of exemption from fasting.

### Statistical analysis

Data were analysed using Stata Version 12.1 SE [9]. Descriptive relationships between demographic variables and fasting behaviour were explored using means, medians and ranges were used for continuous variables, whilst categorical variables were described using proportions. Logistic regression modelling was used to investigate the relationship between the decision to fast or not and the demographic variables. The relationship between fasting behaviours and birth outcomes, including preterm birth, birth weight and low birth weight were examined using logistic regression and linear regression respectively. For the analyses conducted using fasting categories we examined relationships between covariables and birth outcomes using multinomial logistic regression using non-fasters as the reference category. For all final models covariables were screened using univariate analysis and entered into final models if they were significant (p < 0.2). This study had 60% power at a 95% level of confidence to detect a 10% difference in low birth weight and preterm birth. The equivalent power value for differences in birth weight, between fasting and non-fasting mothers, was 90% [10].

## Results

Approximately forty three per cent (n = 128), of all pregnant women reported fasting during Ramadan in 2010. Of these women who fasted, this was undertaken partially by 28 per cent and for the full period of Ramadan by 14 per cent of pregnant women. Of the cohort who chose to fast, most were fasting in their first (n = 42, 32.8%) and second trimesters of pregnancy (n = 74, 57.8%). Table 1 describes the differences in maternal characteristics by fasting behaviour.

**Table 1:** **Maternal characteristics stratified by maternal fasting category and in total+**

	None (n = 172)		Fasted (n = 128)	
Mothers age (years), mean SD	29.0	5.4	27.6	4.7
range	24-32.5		24-30	
**Trimester exposed to fasting**				
1^st^	-		42	32.8
2^nd^	-		74	57.8
3^rd^	-		11	8.6
**Duration of fasting**				
None	172	100		
1-9 days			26	20.3
10-19 days			26	20.3
20-29 days			76	59.4
Total period of Ramadan (29 days)			43	14.3
**Ethnic group**				
Pakistani	161	93.6	114	89.1
Indian	7	4.1	3	2.3
Bangladeshi	4	2.3	9	7.0
Other South Asian	0	0	2	1.6
**Booking BMI category**				
Underweight	10	5.8	10	7.8
Normal	69	40.1	37	28.9
Overweight	46	26.7	31	24.2
Obese	29	16.9	38	29.7
Missing	18	10.5	12	9.4
**Parity**				
0	62	36.0	30	23.4
1	43	25.0	37	28.9
2	28	16.3	24	18.8
3+	35	20.4	33	25.8
Missing	4	2.3	4	3.1
**Marital status**				
Married	163	94.7	127	99.2
Single	4	2.3	1	0.8
Divorced/Separated	5	2.9	0	0
**Maternal employment**				
Currently working	50	29.1	25	19.5
Ever worked	45	26.2	27	21.1
Never worked	77	44.8	76	59.4
**Mothers education**				
<5 GCSE equivalent	32	18.6	39	30.5
5 GCSE equivalent	45	26.2	39	30.5
A-level equivalent	27	15.7	21	16.4
Higher than A-level	63	36.6	27	21.1
Other	5	2.9	2	1.6
**Mothers lives in extended family**				
No	93	54.1	64	50.0
Yes	79	45.9	64	50.0
**Maternal smoking status during pregnancy**				
No	164	95.4	85	100
Yes	8	4.6	0	0
**Mother in consanguineous relationship**				
No	90	52.3	47	36.7
Yes	82	47.7	81	63.3
**Mother born in UK or moved young**				
No	73	42.4	69	53.9
Yes	99	57.6	59	46.1
Gestational diabetes*				
No	149	86.6	108	84.4
Yes	23	13.4	20	15.6
Pregnancy induced hypertension*				
No	156	90.7	111	86.7
Mild to moderate	3	1.7	4	3.1
Severe	1	0.6	1	0.8
Missing	12	7.0	12	9.4
Pre-eclampsia*				
No	157	91.3	116	90.6
Yes	3	1.7	0	0
Missing	12	7.0	12	9.4
***Birth outcomes***				
Birthweight (grams), (SD)	3133	467.4	3219.3	534.4
Gestational length (weeks), SD	39.1	1.6	39.2	1.5
Preterm delivery	6	3.5	4	3.1
Low birth weight (<2500 g), (SD)	14	8.1	8	6.3
Male gender	79	45.9	65	50.8

+ The results of all variables are n (%) unless otherwise stated.

*No existing diabetics, Only 1 participant had pre-existing hypertension.

Next the univariate and multivariate relationship of covariables and fasting behaviours were investigated with non-fasters comprising the reference category. These relationships are shown below in Table 2.

**Table 2:** **Unadjusted and adjusted relationship of covariables to fasting behaviour (Odds compared to non-fasters)**

	Unadjusted results*			Adjusted results		
	Odds Ratio	95% CI	p-value	Odds Ratio	95% CI	p-value
**Mothers age**	**0.95**	**0.90-0.99**	**0.02**	**0.87**	**0.80-0.94**	**<0.01**
**Ethnic group**						
Pakistani	1 (ref)			1 (ref)		
Indian	0.61	0.15-2.39	0.47	1.89	0.38-9.47	0.44
Bangladeshi	3.18	0.96-10.57	0.06	3.77	1.04-13.65	0.04
Other South Asian	-	-				
**Booking BMI category**						
Underweight	1.86	0.64-5.39	0.26	1.89	0.62-5.79	0.27
Normal	1			1		
Overweight	1.26	0.68-2.30	0.41	1.41	0.71-2.81	0.33
Obese	2.44	1.31-4.57	0.03	2.78	1.29-5.97	<0.01
**Parity**						
0	1			1		
1	1.78	0.96-3.30	0.06	2.09	0.98-4.45	0.06
2	1.77	0.88-3.56	0.11	3.69	1.38-9.86	<0.01
3+	1.95	1.02-3.71	0.04	3.99	1.39-11.49	0.01
**Migration status**						
Born in the UK or moved < 5 years	1			1		
Moved to UK >5 years	1.59	1.00-2.51	0.05	1.37	0.73-2.58	0.33
**Maternal employment**						
Currently working	1			1		
Ever worked	1.20	0.61-2.36	0.60	0.80	0.33-1.93	0.62
Never worked	1.54	1.11-3.51	0.02	1.12	0.50-2.51	0.79
**Mothers education**						
<5 GCSE equivalent	1	1			1	
5 GCSE equivalent	0.71	0.38-1.34	0.47	0.63	0.30-1.35	0.23
A-level equivalent	0.64	0.31-1.33	0.42	0.70	0.28-1.76	0.45
Higher than A-level	0.35	0.18-0.67	<0.01	0.40	0.18-0.91	0.03
Other	0.33	0.06-1.81	0.23	0.77	0.11-5.34	0.79
**Mother in consanguineous relationship**						
No		1		1	1	
Yes	1.89	1.19-3.02	<0.01	1.37	0.77-2.51	0.28

*marital status, living in extended family, gestational diabetes and pregnancy induced hypertension were examined but were not found to be associated with fasting in univariate analyses.

The results of univariate analyses showed a higher odds of fasting for women with an obese BMI at booking compared to women of a normal BMI OR 2.44 (95% C.I. 1.31-4.57), women who moved to the UK aged over five compared to women who moved prior to age five or were born in the UK, OR 1.59 (1.00-2.51), higher in women who were in a consanguineous relationship compared to those not in consanguineous relationships, OR 1.89 (95% C.I. 1.19-3.02), and a higher odds of fasting with parity of three or greater compared with nulliparous women OR 1.95 (95% C.I. 1.02-3.71), whereas women who had educational qualifications of greater than A level were less likely to fast compared to women with less than 5 GCSEs, OR 0.35 (95% C.I. 0.18-0.67) as were older women compared to younger women OR 0.95 (95% C.I. 0.90-0.99).

Adjusted analyses were conducted including all variables where covariates had p values greater than 0.2. In these adjusted analyses higher maternal age, OR 0.87 (0.80-0.94), and highest educational qualification greater than A level OR 0.40 (0.18-0.91) remained significantly associated with lower odds of fasting. Likewise higher parity and obese BMI remained significantly associated with higher odds of fasting. Women with a parity of either two or three or greater had a higher odds of fasting, OR 3.69 (95 C.I. 1.38-9.86) and OR 3.99 (95 C.I. 1.39-11.49) respectively, compared to women who were nulliparous and women with an obese BMI had an odds of fasting 2.78 (95% C.I. 1.29-5.97) that of women with a normal BMI at booking. In this final adjusted analyses maternal employment, migration status and consanguinity were no longer significantly associated with odds of fasting. Bangladeshi ethnicity was however found to be associated with higher odds of fasting 3.77 (95% C.I. 1.04-13.65).

We also examined the relationship between fasting categories (day of fasting) and covariables, (Additional file 1: Table S1 and S2) in both unadjusted and adjusted multinomial logistic models. These models were consistent in finding associations between maternal age, booking BMI and parity and the decision to fast during Ramadan whilst pregnant. Educational level was however not associated with a particular fasting duration, whilst being in a consanguineous relationship was associated with an increased odds of being in a 10–19 day fasting duration compared to not fasting.

The birth outcomes, in Table 3 below, showed that no association was observed between fasting and preterm birth, low birth weight, birth weight either before or after adjustment for other covariables.

**Table 3:** **Relationship of preterm birth, low birth weight and birth weight with fasting**

	Unadjusted		Adjusted*	
Outcome	OR (95% CI)	p value	OR (95% CI)	p value
Preterm birth	1.12 (0.31-4.06)	0.86	0.72 (0.12-4.19)	0.71
Low birth weight	0.75 (0.31-1.9)	0.54	1.82 (0.65-5.06)	0.25
Birth weight, mean difference, grams (SD)	86.02 (-28.16-200.20)	0.14	75.07 (-221.27-371.41)	0.62

*All models adjusted for age, fasting, trimester of exposure to fasting, maternal education, parity, booking BMI, height, baby’s gender and smoking.

Finally we examined the association between fasting duration and birth outcomes, the results of which can be seen below in Table 4. No relationship was observed between any category of fasting duration and any of the birth outcomes examined.

**Table 4:** **Relationship of preterm birth, low birth weight and birth weight with fasting**

	Unadjusted		Adjusted*	
Preterm birth	OR (95% CI)	p value	OR (95% CI)	p value
None	1		1	
1-9 days	0.90 (0.10-7.82)	0.93	-	
10-19 days	0.90 (0.10-7.82)	0.93	0.68 (0.1-10.7)	0.79
20-29 days	1.33 (0.26-6.78)	0.73	0.22 (0.1-1.8)	0.16
**Low birth weight**				
None	1		1	
1-9 days	0.45 (0.06-3.59)	0.45	1.04 (0.11-9.59)	0.97
10-19 days	0.45 (0.06-3.59)	0.45	1.40 (0.15-13.26)	0.77
20-29 days	0.97 (0.36-2.62)	0.95	2.23 (0.71-6.29)	0.17
**Birth weight, mean difference, grams(SD)**				
None	1		1	
1-9 days	165.94 (-40.16-2-372.06)	0.11	150.89 (-201.07-502.85)	0.40
10-19 days	115.2 (-90.93-321.29)	0.27	68.41 (-252.83-389.66)	0.67
20-29 days	48.7 (-86.22-183.62)	0.48	45.61 (-276.39-367.62)	0.78

*All models adjusted for age, fasting, trimester of exposure to fasting, maternal education, parity, booking BMI, height, baby’s gender and smoking.

## Discussion

### Main findings

Over forty per cent of pregnant British Asian Muslim women in this study were found to have fasted for at least one day during Ramadan. Complete fasting for the entire Ramadan period was not common, reported by only 14 per cent of women and most fasting occurred during the first and second trimester of pregnancy.

The decision to fast during pregnancy was found to be negatively associated with mother’s age and educational level, as both age and education levels increased, the odds of fasting reduced. Factors increasing the odds of fasting during pregnancy included an obese BMI at booking, parity of two or more and Bangladeshi ethnic origin. When individual categories of fasting behaviour were examined both parity and obese BMI at booking were associated with increasing duration whereas educational level was not. Finally, we found no association between any maternal health measures, such as gestational diabetes or hypertension during pregnancy and fasting behaviours. It remains unclear if pregnant women with health problems such as gestational diabetes or pregnancy induced hypertension did not wish to take up the medical exemption from fasting or were fasting in trimesters prior to finding out about these health problems.

We found no association between the decisions to fast or not and the two birth outcomes examined; preterm birth or mean birth weight, either when examined as crude associations or after adjustment for covariables. Additionally, we found no evidence of a dose response relationship between duration of fasting in days and either of the birth outcomes examined.

### Strengths and limitations

The major strength of this study is that it contains results from a large contemporary sample of pregnant South Asian Muslim women in the United Kingdom and contains self-reported measures of their fasting behaviours during the Ramadan period in addition to a wide range of socio-demographic characteristics. A literature search conducted in PubMed over the time period 1966 to 2014 found no results of studies that explored the characteristics or fasting behaviours of UK resident pregnant Muslim women. Our study is therefore the first undertaken in the UK to our knowledge that attempts to describe differences in fasting behaviours during pregnancy according to maternal characteristics. The findings of these analyses have been adjusted for covariables hypothesised to be associated with fasting to ensure that findings are robust. For our secondary outcomes of preterm birth and birth weight we have also been able to adjust for potential confounders, such as pre-existing health problems additionally clinical covariables such as parity and length of gestation were available from clinical notes ensuring these data were not subject to recall bias.

Most studies reporting negative impacts of prenatal Ramadan fasting were conducted without access to reported fasting data, so have been unable to examine the potential role of duration of exposure nor had they accurately recorded the timing of exposure to Ramadan using ultrasound data or other objective measures [2, 11, 12] with the exception of a substudy included in one of the studies [2]. Finally, these studies have not been able to examine the potential bias that may exist if, as our study has found, there are systematic differences in maternal characteristics vary of women with different fasting behaviours during Ramadan.

One limitation of our study was our period of recruitment, at 26–28 weeks gestation, which may mean that we missed earlier preterm deliveries. Most preterm births are however likely to occur after 28 weeks, meaning that our results are a conservative estimate of Ramadan fasting exposure [13]. A further limitation may be the external generalizability of these results to the wider UK Muslim populations which may comprise different ethnic groups with varying traditions and beliefs about fasting. Despite this limitation 2011 England census figures show that over 68 per cent of all UK Muslims were from a South Asian ethnic background suggesting that our data will be largely generalizable [14]. Another limitation of our study is that we have a small proportion of women who fasted during the third trimester. A further limitation of our study is related to our sample size which may be underpowered to find evidence of a relationship between fasting behaviours and birth outcomes should this exist.

### Interpretation in light of other findings

Our results, showing that pregnant women who are fasting are heavier and have a higher parity, have also been reported in studies conducted in Iran and Singapore respectively [15, 16]. The relationships we observed between higher parity and lower maternal age being related to fasting were however not observed in another Iranian maternal cohort [17]. Our results on the relationship between educational qualifications and fasting behaviours of mothers have not been previously reported. Most of the women included in our cohort were fasting during their first and second trimesters of pregnancy. Third trimester exposure to low calorific fasting has been shown to result in lower than average birth weights [18], although data from exposure to Ramadan fasting by trimester suggests exposure to fasting at any point in pregnancy may be associated with birth outcomes whereas adult outcomes may be more affected by fasting early in pregnancy [2].

Although it has been reported that around 70–90 per cent of all pregnant Muslim women fast during pregnancy [2] only 40 to 55 per cent of these will fast for the entire Ramadan period [15, 17]. Results from maternal populations in Pakistan, the largest country of origin for cohort participants, found complete fasting rates of 42 per cent [19]. Our results from the UK showing lower rates of maternal fasting may also be further influenced by geography, as the duration of the fast locally during the summer months is longer than that experienced by participants in Middle Eastern and South East Asian locations where these other studies were conducted.

These results from these studies indicate that studies examining relationships between fasting behaviour and infant and later health outcomes may produce biased findings if it is assumed that behaviours are homogenous amongst pregnant Muslim populations in the UK. Our results indicate there are systematic differences in fasting behaviours between pregnant women by booking BMI and parity, factors which are known to be causally related to birth outcomes such as birth weight and preterm birth [20, 21].

Our studies are consistent with others in not finding an association between fasting and preterm delivery [8]. Associations between fasting during Ramadan and birth weight have been inconsistently observed in previous research with some studies finding an association [17] and others not [3, 4, 16, 22].

## Conclusions

Our results indicate that just under half of all pregnant Muslim women living in the UK fast, although most do not do so for full period of Ramadan. Women fasting for the full period of Ramadan exhibit different socio-cultural characteristics than their non-fasting peers. Studies examining long term impacts of fasting behaviours of pregnant Muslim women without access to objective fasting behaviour may introduce bias to their results if fasting behaviours are assumed to be homogenous. Further studies are required to replicate our findings and larger studies with data on actual fasting behaviours are required to provide further evidence on the dose response relationship of fasting and to enable investigation of the effects of fasting by trimester of exposure.

### Details of ethics approval

The participants gave informed consent for the data collection and ethical approval for the data collection was granted by Bradford Research Ethics Committee (Ref 07/H1302/112).

Born in Bradford is only possible because of the enthusiasm and commitment of the Children and Parents in BiB. We are grateful to all the participants, health professionals and researchers who have made Born in Bradford happen.

## Electronic supplementary material

Additional file 1: Table S1.: Unadjusted relationship of covariables to fasting behaviour. **Table S2.** Adjusted relationship of covariables to fasting behaviour. (DOC 98 KB)
