# Supplementary material for: Experiences and outcomes of maternal Ramadan fasting during pregnancy: results from a sub-cohort of the Born in Bradford birth cohort study
Source: BMC Pregnancy Childbirth. 2014 Sep 26;14:335. doi: 10.1186/1471-2393-14-335 (PMC4261761; doi:10.1186/1471-2393-14-335)
Supplement: Supplementary file 1 — Additional file 1: Table S1.: Unadjusted relationship of covariables to fasting behaviour. Table S2. Adjusted relationship of covariables to fasting behaviour. (DOC 98 KB) [file 12884_2014_1247_MOESM1_ESM.doc]

Supplementary tables

Supplementary material

**Table S1 Unadjusted relationship of covariables to fasting behaviour**

|  | **Fasting duration categories (compared to non-fasters)** | | | | | |
| --- | --- | --- | --- | --- | --- | --- |
|  | **1-9 days** | | **10-19 days** | | **20-29 days** | |
|  | **OR (95% CI)** | **P** | **OR (95% CI)** | **P** | **OR (95% CI)** | **P** |
| Mothers age, years | 1.0 (0.9-1.1) | 0.93 | 0.9 (0.8-0.9) | 0.01 | 0.9 (0.9-1.0) | 0.05 |
| Booking BMI category, n(%) |  |  |  |  |  |  |
| Underweight | 0.8 (0.1-6.7) | 0.81 | 3.5 (0.9-13.6) | 0.08 | 1.7 (0.5-5.6) | 0.37 |
| Normal | 1 |  | 1 |  | 1 |  |
| Overweight | 1 (0.3-3.0) | 1.00 | 1.1 (0.4-3.5) | 0.84 | 1.4 (0.7-3.0) | 0.34 |
| Obese | 2.6 (0.9-7.2) | 0.06 | 2.4 (0.8-6.9) | 0.11 | 2.4 (1.1-5.1) | 0.03 |
| Parity |  |  |  |  |  |  |
| 0 | 1 |  | 1 |  | 1 |  |
| 1 | 1.2 (0.4-3.9) | 0.72 | 2.2 (0.7-6.5) | 0.17 | 1.9 (0.9-3.9) | 0.10 |
| 2 | 1.6 (0.5-5.4) | 0.47 | 1.5 (0.4-5.6) | 0.57 | 1.9 (0.9-4.5) | 0.11 |
| 3+ | 1.7 (0.6-5.5) | 0.32 | 1.2 (0.3-4.5) | 0.81 | 2.3 (1.1-4.9) | 0.03 |
| Migration status |  |  |  |  |  |  |
| Born in the UK or moved<5 years | 1 |  |  |  |  |  |
| Moved to UK >5 years | 1.4 (0.6-3.1) | 0.47 | 1.0 (0.4-2.3) | 0.99 | 2.0 (1.1-3.4) | 0.02 |
| Maternal employment |  |  |  |  |  |  |
| Currently working | 1 |  | 1 |  | 1 |  |
| Ever worked | 1.9 (0.5-7.1) | 0.31 | 1.5 (0.5-4.6) | 0.50 | 0.9 (0.4-2.1) | 0.79 |
| Never worked | 2.4 (0.8-7.8) | 0.13 | 1.3 (0.5-3.7) | 0.62 | 2.1 (1.1-4.2) | 0.03 |
| Maternal education |  |  |  |  |  |  |
| <5 GCSE equivalent | 1 |  | 1 |  | 1 |  |
| 5 GCSE equivalent | 0.2 (0.1-0.8) | 0.03 | 1.0 (0.4-2.7) | 0.97 | 0.9 (0.4-1.8) | 0.66 |
| A-level equivalent | 0.6 (0.2-1.9) | 0.38 | 0.3 (0.1-1.50 | 0.14 | 0.8 (0.3-1.8) | 0.59 |
| Higher than A-level | 0.4 (0.1-1.1) | 0.08 | 0.3 (0.1-0.9) | 0.04 | 0.4 (0.2-0.80) | 0.01 |
| Other | - |  | 0.8 (0.1-7.8) | 0.85 | 0.3 (0.1-2.8) | 0.29 |
| Consanguineous relationship |  |  |  |  |  |  |
| No | 1 |  | 1 |  | 1 |  |
| Yes | 1.5 (0.7-3.4) | 0.34 | 6.0 (2.0-18.3) | >0.01 | 1.5 (0.9-2.6) | 0.14 |
| Live in extended family |  |  |  |  |  |  |
| No | 1 |  | 1 |  | 1 |  |
| Yes | 1.0 (0.4-2.3) | 0.98 | 1.2 (0.5-2.7) | 0.70 | 1.2 (0.7-2.1) | 0.43 |

**Table S2 Adjusted relationship of covariables to fasting behaviour**

|  | **Fasting duration categories (compared to non-fasters)** | | | | | |
| --- | --- | --- | --- | --- | --- | --- |
|  | **1-9 days** | | **10-19 days** | | **29 days** | |
|  | **OR (95% CI)** | **P** | **OR (95% CI)** | **P** | **OR (95% CI)** | **P** |
| Mothers age | 0.9 (0.8-1.1) | 0.32 | 0.8 (0.7-0.9) | >0.01 | 0.9 (0.8-0.9) | >0.01 |
| Booking BMI category, n(%) |  |  |  |  |  |  |
| Underweight | 0.9 (0.1-9.7) | 0.99 | 2.9 (0.6-15.4) | 0.2 | 2.4 (0.9-5.5) | 0.08 |
| Normal | 1 |  | 1 | 1 | 1 |  |
| Overweight | 1.2 (0.4-3.7) | 0.80 | 0.9 (0.2-3.5) | 0.90 | 1.6 (0.7-3.5) | 0.28 |
| Obese | 3.9 (1.2-12.7) | 0.02 | 2.2 (0.5-8.7) | 0.27 | 3.0 (1.2-7.2) | 0.02 |
| Parity |  |  |  |  |  |  |
| 0 | 1 |  | 1 |  | 1 |  |
| 1 | 0.2 (0.1-1.5) | 0.98 | 2.8 (0.8-10.3) | 0.13 | 2.2 (0.9-5.5) | 0.08 |
| 2 | 0.1 (0.1-1.6) | 0.50 | 3.8 (0.6-23.9) | 0.15 | 4.8 (1.6-15.1) | >0.01 |
| 3+ | - | - | 2.5 (0.3-21.1) | 0.41 | 5.5 (1.6-19.1) | >0.01 |
| Migration status |  |  |  |  |  |  |
| Born in the UK or moved<5 years | 1 |  | 1 |  | 1 |  |
| Moved to UK >5 years | 1.1 (0.4-3.2) | 0.80 | 0.7 (0.2-2.1) | 0.54 | 1.8 (0.8-3.7) | 0.13 |
| Maternal employment |  |  |  |  |  |  |
| Currently working | 1 |  | 1 |  | 1 |  |
| Ever worked | 1.8 (0.4-7.8) | 0.41 | 1.4 (0.3-6.8) | 0.69 | 0.6 (0.2-1.6) | 0.29 |
| Never worked | 2.1 (0.5-8.4) | 0.30 | 0.7 (0.2-3.2) | 0.65 | 1.1 (0.4-2.7) | 0.91 |
| Maternal education |  |  |  |  |  |  |
| <5 GCSE equivalent | 1 |  |  | 1 | 1 |  |
| 5 GCSE equivalent | 0.2 (0.1-0.8) | 0.02 | 0.6 (0.2-2.1) | 0.46 | 0.8 (0.3-2.0) | 0.66 |
| A-level equivalent | 0.7 (0.2-2.8) | 0.61 | 0.1 (0.1-2.1) | 0.04 | 1.18 (0.4-3.4) | 0.76 |
| Higher than A-level | 0.5 (0.2-1.7) | 0.27 | 0.3 (0.1-1.3) | 0.10 | 0.4 (0.2-1.2) | 0.10 |
| Other | - |  | 0.8 (0.1-13.8) | 0.87 | 1.1 (0.1-12.5) | 0.93 |
| Consanguineous relationship |  |  |  |  |  |  |
| No | 1 |  | 1 |  | 1 |  |
| Yes | 1.1 (0.4-3.0) | 0.80 | 4.1 (1.2-14.5) | 0.03 | 0.9 (0.5-1.8) | 0.78 |
